# Exploring trends in admissions and treatment for ankle fractures: a longitudinal cohort study of routinely collected hospital data in England

**DOI:** 10.1186/s12913-020-05682-9

**Published:** 2020-08-31

**Authors:** Lauren J. Scott, Tim Jones, Michael R. Whitehouse, Peter W. Robinson, William Hollingworth

**Affiliations:** 1 NIHR ARC West, University Hospitals Bristol and Weston NHS Foundation Trust, 9th Floor Whitefriars, Lewins Mead, Bristol, BS1 2NT UK; 2 Department of Population Health Sciences, Bristol Medical School, University of Bristol, Bristol, BS8 2PS UK; 3 Musculoskeletal Research Unit, Translational Health Sciences, Bristol Medical School, 1st Floor Learning & Research Building, Southmead Hospital, Bristol, BS10 5NB UK; 4 National Institute for Health Research Bristol Biomedical Research Centre, University Hospitals Bristol and Weston NHS Foundation Trust and University of Bristol, Bristol, UK; 5 Avon Orthopaedic Centre, Southmead Hospital, North Bristol NHS Trust, Westbury-on-Trym, Bristol, BS10 5NB UK

**Keywords:** Ankle fracture, Hospital admission, England, Intramedullary fixation, Extramedullary fixation, Close-contact-casting

## Abstract

**Background:**

Evidence on the most effective and cost-effective management of ankle fractures is sparse but evolving. A recent large RCT in older patients with unstable fractures found that management with close-contact-casting was functionally equivalent and more cost-effective than internal fixation. We describe temporal and geographic variation in ankle fracture management and estimate the potential savings if close-contact-casting was used more often in older patients.

**Methods:**

Patients admitted to hospital in England between 2007/08 and 2016/17 with an ankle fracture were identified using routine hospital episode statistics. We tested whether the use of internal fixation, and the proportion of internal fixations using intramedullary implants, changed over time. We estimated the potential annual cost savings if patients aged 60+ years were treated with close-contact-casting rather than internal fixation, in line with emerging evidence.

**Results:**

Over the 10-year period, there were 223,465 hospital admissions with a primary ankle fracture diagnosis. The incidence (per 100,000) of internal fixation was fairly consistent over time in younger (33.2 in 2007/08, 30.9 in 2016/17) and older (36.5 in 2007/08, 37.4 in 2016/17) patients. The proportion of internal fixations which used intramedullary implants increased in both age groups (17.0–19.5% < 60 years; 15.2–17.4% 60+ years). In 2016/17, the cost of inpatient hospital care for ankle fractures in England was over £63.1million. If 50% of older patients who had an internal fixation instead had close-contact-casting, we estimate that approximately £1.56million could have been saved.

**Conclusions:**

Despite emerging evidence that non-surgical and surgical management achieve equivalent functional outcomes in older patients, the rate of surgical fixation has remained relatively stable over the decade. The health service could achieve substantial savings if a higher proportion of older patients were treated with close-contact-casting, in line with recent evidence.

## Background

Ankle fractures are increasingly common in the UK [1]. Trauma and frailty both play a role in the aetiology, with peaks in younger men and older women [1, 2]. As costs of fractures are high [3], it is important to identify and implement cost-effective care and to reduce variation in the care delivered.

In 2012, a systematic review [4] concluded there was insufficient evidence regarding the appropriateness of surgery versus conservative treatment of ankle fractures. Subsequently, two randomised controlled trials (RCTs) [5, 6] in younger adults with lateral malleolus fractures reported equivalent functional outcomes following internal surgical fixation versus non-surgical (cast or brace [6], boot [5]) treatment at 12-months. Participants in the surgical group spent longer in hospital and had more minor adverse events [5], however, delayed bone union was more common in the non-surgical group [6]. In older patients with unstable ankle fractures, a large RCT comparing internal fixation versus close-contact-casting reported equivalent ankle function at 6-months [7]. Infections and additional procedures were more common in the surgical group (10% vs. 1 and 6% vs. 1%, respectively), although 19% of patients initially treated with casts later had surgery. A higher rate of malunion and non-union of the medial malleolus was observed at long-term follow-up in the close-contact-cast group but this did not affect functional outcomes which remained equivalent at 3 years [7, 8]. Close-contact-casting resulted in high initial and modest long-term cost savings and had a high probability of being cost-effective [9].

Small trials have evaluated the role of intramedullary implants compared to extramedullary fixation for lateral malleolar [10], bimalleolar or trimalleolar fractures [11, 12]. These trials indicate similar [10, 12] or better [11] functional outcomes in the intramedullary groups with fewer complications [10–12], which may offset the higher initial cost of the implant [10].

The 2016 British Orthopaedic Association Standards for Trauma and Orthopaedics (BOAST) 12 guidelines recommend early surgical fixation for unstable ankle fractures in patients < 60 years, and suggest close-contact-casting may be an option in patients over 60 years [13]. Close-contact-casting is applied in an operating theatre under general or spinal anaesthetic by an orthopaedic surgeon. This differs from a standard plaster cast which can be applied without anaesthetic in an outpatient environment [7].

### Aims

To describe age, sex, temporal and geographic variation in ankle fracture admissions and management in England using routine Hospital Episode Statistics data. We estimated the costs of these admissions and discuss the potential impact of adopting recent evidence into clinical practice on those costs.

## Patients and methods

### Data source

Ankle fractures were identified from hospital episode statistics (HES) admitted patient care data. HES records all patient care episodes funded by the NHS [14, 15]. An episode is defined as a period of care under one consultant team; therefore, a hospital admission may contain multiple episodes. International Classification of Diseases version 10 (ICD10) codes are used to record a primary and up to 19 additional diagnoses. Up to 24 procedures (Office of Population, Censuses and Surveys [OPCS] procedure codes) and demographic data including patient sex, age and area of residence are recorded for each episode. HES data is primarily collected for administrative purposes, including hospital reimbursement, but is often used for research. This study included adults (aged 16+ years) admitted between 1st April 2007 and 31st March 2017. We restricted our analyses to patients with inpatient admissions to hospital, as diagnoses are poorly recorded in emergency and outpatient department HES datasets.

### Identifying index admission and readmission of ankle fractures

**Fig. 1:**
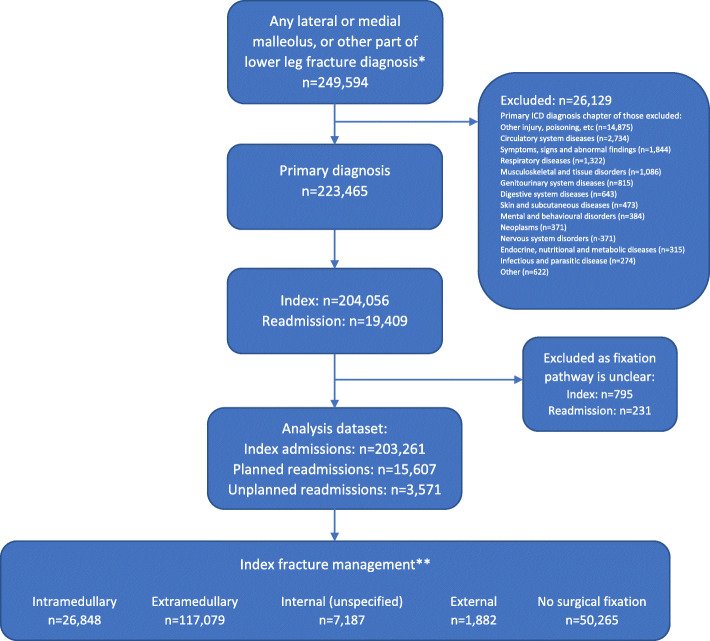
Patient flowchart. *Patients with primary diagnoses of fractures of the lower end of tibia (S823; *n* = 49,478), fibular alone (S824; *n* = 32,609), and lower leg part unspecified (S829; *n* = 730) were not considered for this analysis as codes were not sufficiently specific. ** 1560 intramedullary, 6941 extramedullary, 500 internal (unspecified), and 112 external fixations occurred during planned readmissions in patients who had no surgical fixation on their index admissions

Patients with diagnosis codes of ‘fracture of medial malleolus’ (S825), ‘fracture of lateral malleolus’ (S826) or ‘fracture of other parts of lower leg’ (S828) as their primary diagnosis on their first episode within an admission were included (Fig. 1). The diagnosis code S828 primarily includes bimalleolar, trimalleolar and Maisonneuve’s fractures, but may also include a small number of pilon (periarticular) fractures [16].

An index admission was defined as the first ankle fracture admission during the study period for a given patient, or an admission which occurred more than 365 days after a previous index admission for the same patient. A readmission was defined as a new ankle fracture admission which occurred 1–365 days following an index admission. Surgeons will sometimes discharge a patient to allow soft tissue swelling to go down and then readmit them, typically within 2–3 weeks, to perform surgery. As such, for this study, readmissions which occurred within 3 weeks of the index admission were assumed to be planned.

### Defining fracture management

Procedure codes were used to define fracture management categories: intramedullary; extramedullary; internal (unspecified); external; and no surgical fixation (supplementary material 1). If more than one surgical fixation occurred within an admission, the first recorded fixation was used to categorise; the exception to this was if someone had an external fixator followed by an internal fixation, then the first internal fixation was used to categorise. As initial non-surgical stabilisation may be followed by planned surgery (as described above), we chose to categorise patients with no surgical fixation on the index admission, but surgical fixation on a readmission within 3 weeks, according to the first surgical fixation received during readmission (Fig. 1). Index admissions and any corresponding readmissions were excluded from the cohort if the OPCS code recorded only adjustment or removal of hardware but there was no code documenting the original fixation (Fig. 1).

### Statistical analyses

Patient demographics are presented as counts and percentages or medians and interquartile ranges (IQRs), as appropriate. Crude admission rates were based on populations from June in the corresponding year [17].

### Temporal trends

We report the annual number of ankle fracture admissions stratified by age group (16–59 and 60+ years). We tested whether the incidence rate of internal fixation changed over the study period in either age group using Poisson regression with a binary age term, a categorical year term and an age-by-year interaction term, with the population in England (of 16+ year olds) in the corresponding year and age group as the offset. To test whether the proportion of internal fixations using intramedullary implants, and the proportion of admission which were day cases, changed over time, we used chi-squared tests for linear trend in each age group.

### Geographic variation

Geographical comparisons were made based on the clinical commissioning group (CCG) in which the patient resided. CCGs are NHS bodies responsible for planning and commissioning health care services for their local area; populations vary in size, covering a median of approximately 250,000 people [17]. As some procedures are relatively rare, we combined the most recent 5 years of data (2012/13–2016/17) to provide more stable estimates of recent procedure rates. We estimated indirectly standardised rate ratios of (a) any surgical fixations and (b) intramedullary fixations, using the population in England in June 2016 as the reference population, adjusting for age (16–39 years, 40–59 years, 60–79 years and 80+ years) and sex [18]. We plot these ratios by CCG (as a percentage of the national average) to evaluate geographic variation in care provided. We converted the indirectly standardised rate ratios to adjusted yearly rates by multiplying them by the crude fixation rate of the reference population [19].

### Treatment costs

The cost to CCGs of inpatient care for ankle fractures, including index admissions and readmissions, were estimated for 2016/17 by linking secondary usage service generated core spell healthcare resource group (HRG) codes for each admission in HES with the 2016/17 payment by results tariff [20, 21]. HRG codes are assigned to each health care spell (i.e. hospital admission) based on their expected resource use; the payment by results tariffs are updated every year to reflect the cost of these resources. Based on evidence that close-contact-casting instead of internal fixation might save approximately £650 per patient [9], we estimated the potential annual savings that might be achieved if arbitrary proportions (25, 50, 75%) of patients aged 60+ years treated with internal fixations were instead treated with close-contact-casting.

All statistical analyses were conducted using Stata/MP 15.1.Variations in procedure rates across England were mapped using ArcGIS ArcMap 10.5.1 for Desktop, Environmental Systems Research Institute, Inc., 2017; the University of Bristol hold an Advanced, Concurrent-use licence for ArcGIS software.

## Results

### Study cohort

Over the 10-year study period, 249,594 hospital admissions included an ankle fracture diagnosis; for 223,465 patients (89.5%), this was the primary diagnosis. After exclusions where the fixation pathway was unclear, our analysis cohort included 203,261 index admissions, 15,607 planned readmissions and 3571 unplanned readmissions (Fig. 1). Forty-two percent of the cohort were male and the median age was 52 years (IQR 35 to 68). Admissions included 52,914 (26%) lateral malleolus fractures, 21,784 (11%) medial malleolus fractures and 128,563 (63%) bimalleolar/trimalleolar/Maisonneuve fractures (Table 1). There were 19,258 admissions for ankle fractures (46.3 per 100,000 people) in 2007/08 and 19,842 (44.4 per 100,000) in 2016/17. Incidence rose steeply with age in females, with 27.3 and 120.8 per 100,000 women aged 16–39 years and 80+ years respectively in 2016/17; incidence in males was higher than females in 16–39 year olds (39.6 per 100,000) but remain reasonably stable with age (Table 2). Over the study period, the percentage of males decreased from 45% in 2007/08 to 40% in 2016/17, and the median age of patients increased from 50 years (IQR 33 to 67) in 2007/08 to 54 years (IQR 36 to 69) in 2016/17.

**Table 1:** Demographics of all patients with an index admission (*n* = 203,261)

	n	%
**Age group**^a^		
16–39 years	62,382	30.7%
40–59 years	61,338	30.2%
60–79 years	56,998	28.0%
80+ years	22,530	11.1%
**Sex**^b^		
Male	86,238	42.4%
Female	117,012	57.6%
**Fracture type**		
Lateral malleolus	52,914	26.0%
Medial malleolus	21,784	10.7%
Bi/tri malleolar or Maisonneuve’s	128,563	63.3%

^a^Data missing for 13 patients. ^b^Data missing for 11 patients

**Table 2:** Incidence of admissions for ankle fractures in 2016/17 by age and sex

	Male		Female		Overall	
Age group	n	n per 100,000 people	n	n per 100,000 people	n	n per 100,000 people
16–39 years	3452	39.6	2331	27.3	5784	33.5
40–59 years	2436	33.6	3499	47.2	5935	40.5
60–79 years	1593	32.7	4119	78.2	5712	56.3
80+ years	450	42.6	1955	120.8	2405	90.0
Overall	7933	36.2	11,908	52.2	19,842	44.4

Note. One person was missing sex data and six people were missing age data and as such are missing from the age/sex specific numbers. However, they are included in the overall numbers where possible, so column and row totals do not quite add up

### Fracture management

**Fig. 2:**
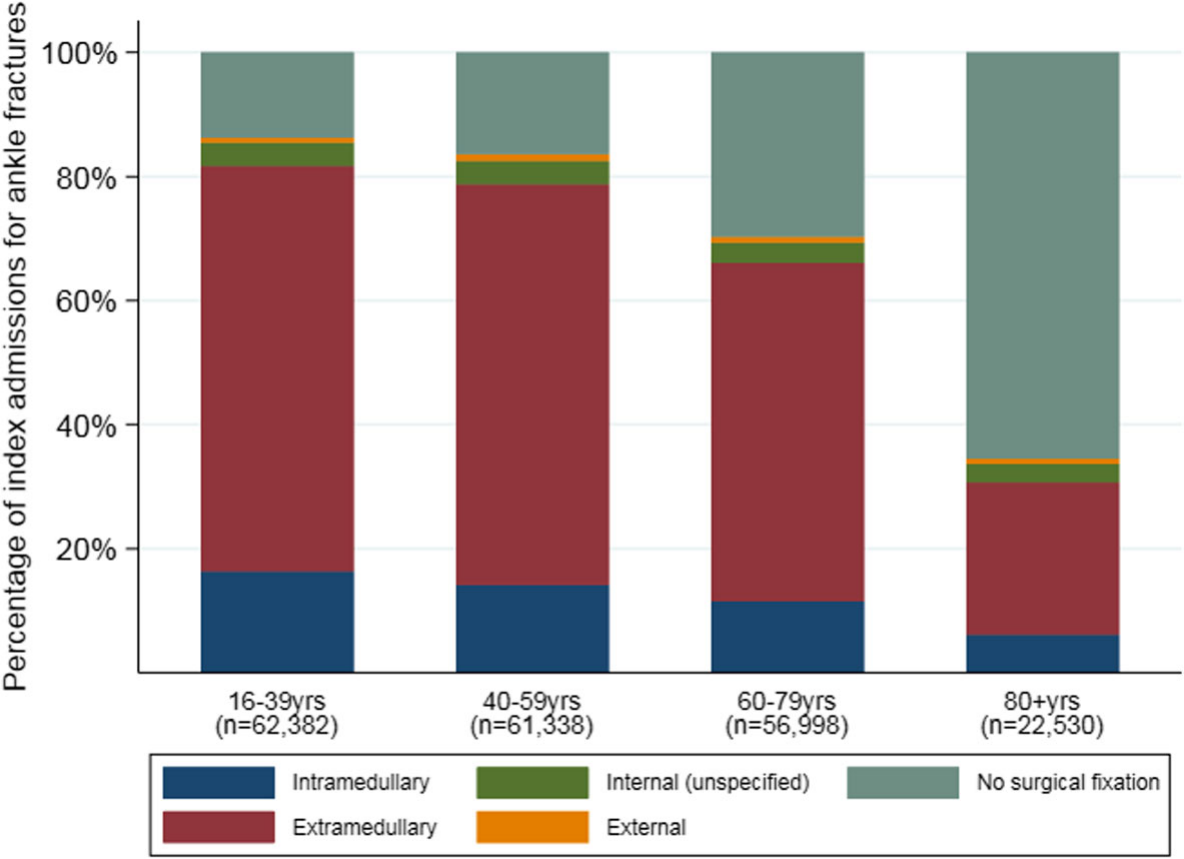
Fracture management by age

**Fig. 3:**
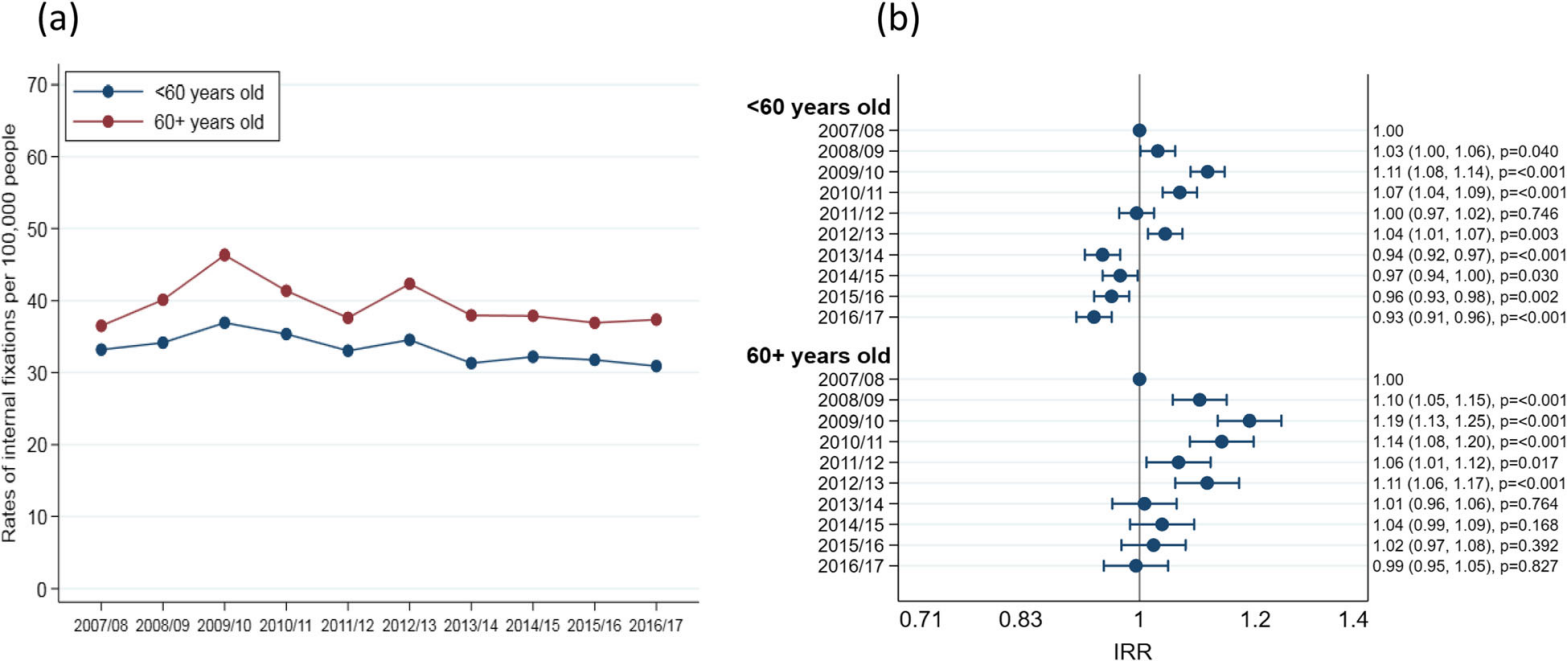
Internal fixations over time by age group. **a** Rates per 100,000 people, **b** Incidence rate ratios (IRRs) from Poisson regression

**Fig. 4:**
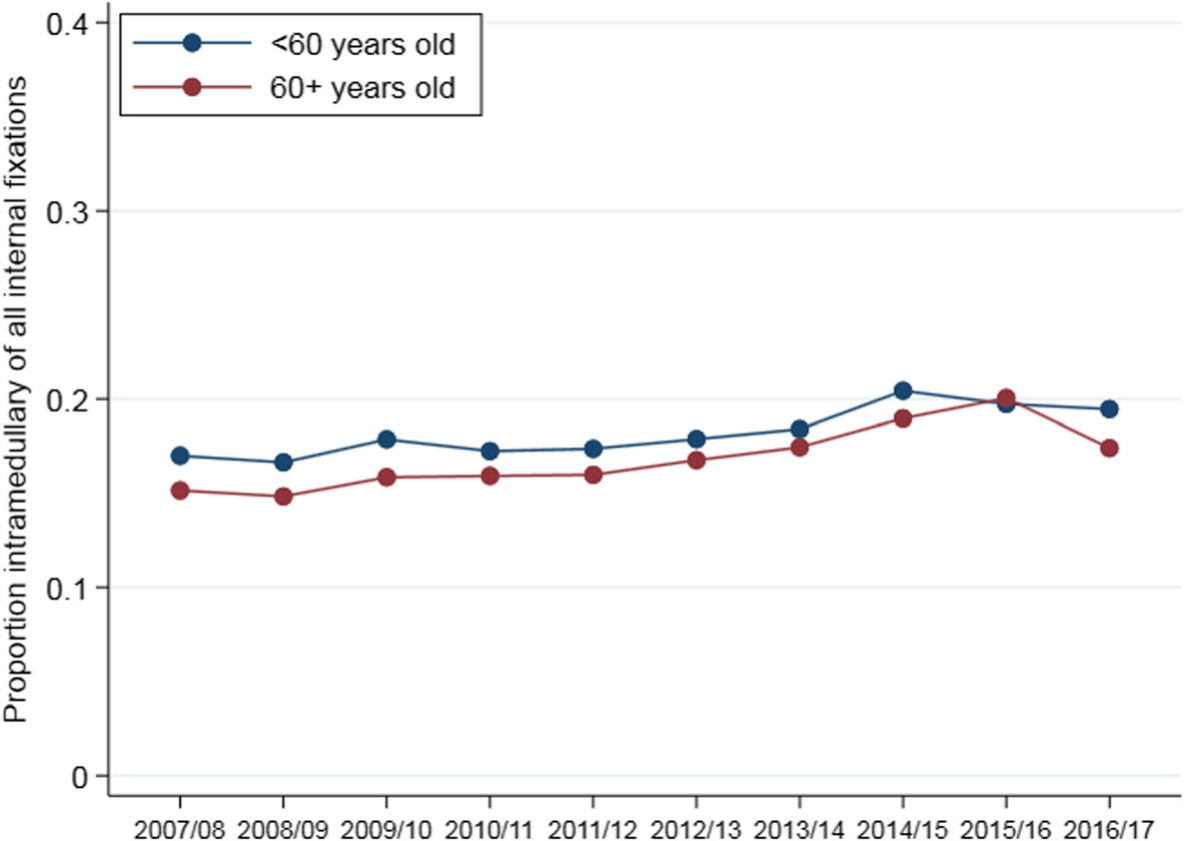
Proportion of internal fixations which are intramedullary over time by age group

**Fig. 5:**
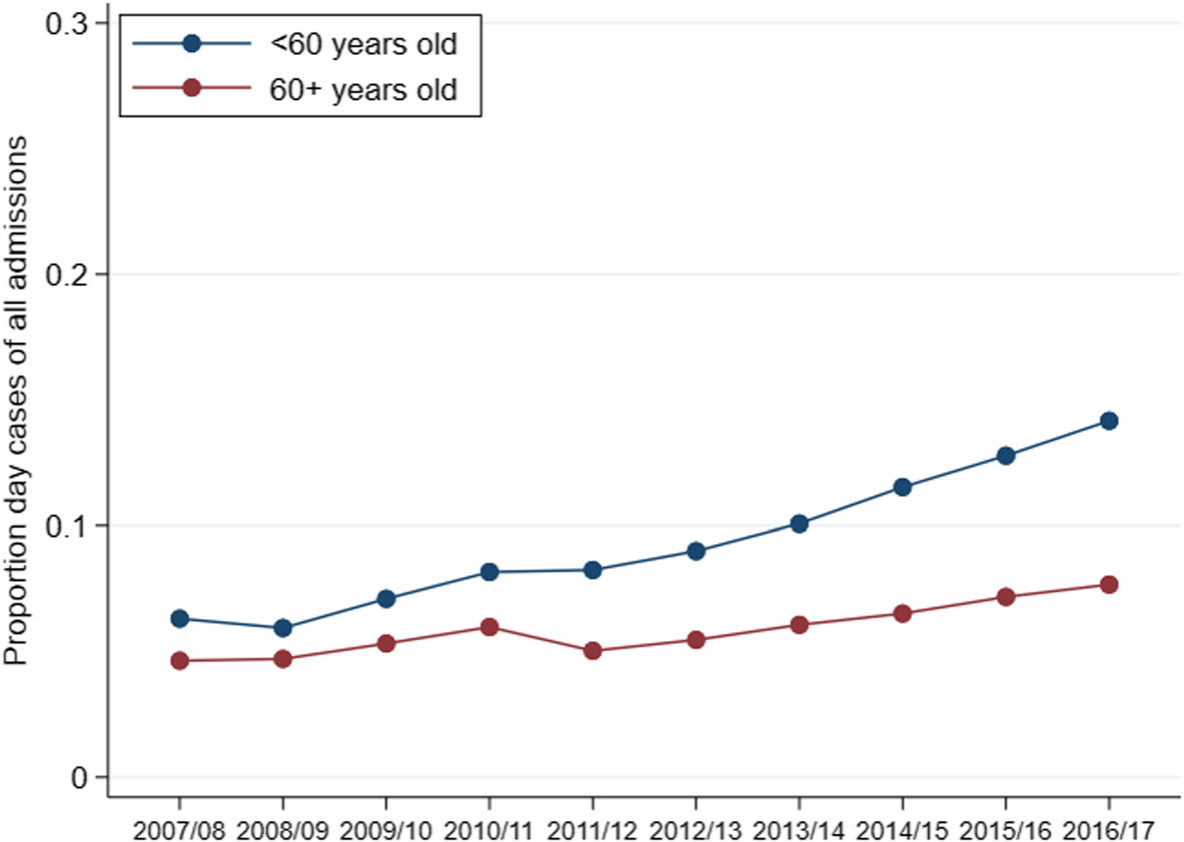
Proportion of admissions which are day cases over time by age group

Management differed by age (Fig. 2). Surgical fixation was far more common in 16–59 year olds (85%) than 80+ year olds (35%). Extramedullary fixations occurred more often than intramedullary fixations in all age groups. The incidence of internal fixation of ankle fractures changed a little over time in both age groups (16–59 and 60+ years), with an increase between 2007/08 and 2009/10 and a generally decreasing trend from 2009/10 onwards (Fig. 3). The proportion of internal fixations using intramedullary implants increased over the study period in both younger (chi2 test for trend 76.2, *p* < 0.001) and older (chi2 test for trend 59.7, p < 0.001) patients, although these changes were relatively small (Fig. 4). The proportion of admissions managed as day cases increased over the study period in both younger (chi2 test for trend 953.3, p < 0.001) and older (chi2 test for trend 111.7, p < 0.001) patients (Fig. 5); this increase was greater in younger patients (6.3% in 2007/08 compared to 14.2% in 2016/17) than older patients (4.6% in 2007/08 compared to 7.7% in 2016/17).

### Geographical variation

**Fig. 6:**
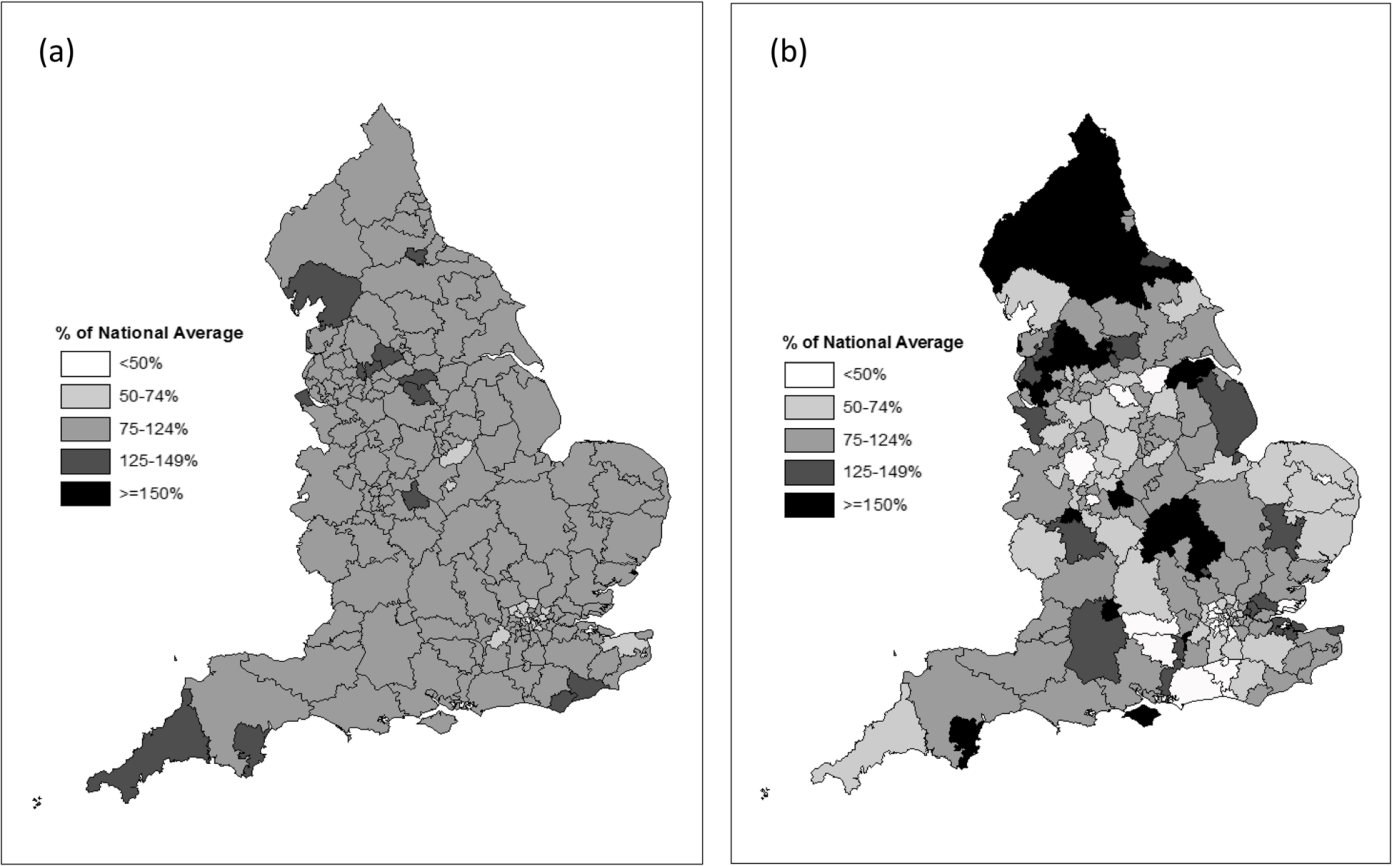
Geographical variation of surgical fixations. **a** Any surgical fixation, **b** Intramedullary fixations. Figures show indirectly standardised rate ratios and include data from 2012/13–2016/17. These figures were created using ArcMap software with the University of Bristol’s advanced, concurrent-use licence for ArcGis software (see methods for details)

After standardising for age and sex, the median yearly incidence rate of surgical fixation across all CCGs was 34.0 per 100,000 people. Figure 6a shows little variation in surgical fixation rates between CCGs across England; incidence in the CCG at the 90th percentile (41.1 per 100,000) was approximately 53% higher (rate ratio [RR] 1.53, 95% confidence interval [19] [CI] 1.30 to 1.78) than the CCG at the 10th percentile (26.9 per 100,000). Intramedullary fixations had relatively larger variation between CCGs (Fig. 6b) with a median of 5.8 per 100,000 people and incidence in the CCG at the 90th percentile (10.9 per 100,000) approximately 3.6 times higher (RR 3.55, 95% CI 2.59 to 4.88) than the CCG at the 10th percentile (3.1 per 100,000).

### Readmissions

Unplanned readmissions occurred following 3217/203,261 (1.6%) index admissions, with 3571 unplanned readmissions in total. This included 1.2% (309/26,848) of intramedullary fixations, 1.0% (1151/117,079) of extramedullary fixations and 2.9% (1461/50,265) of index visits with no surgical fixation. Only 31 (0.1%) intramedullary and 137 (0.1%) extramedullary fixations required readmission for removal. Supplementary material 2 details all fracture management which occurred during these unplanned readmissions. There were also a further 15,607 planned readmissions in 14,628 patients.

### Treatment costs

In 2016/17, the cost to CCGs of inpatient hospital care for ankle fractures in England was more than £63.1million; this included £33.8million and £19.7million spent on internal fixations in patients 16–59 and 60+ years old, respectively. The median cost of care was £3034 (IQR £1969 to £4084) for internal fixations and £1016 (IQR £557 to £1819) for no surgical fixation. If 25, 50% or 75% of older patients (60+ years) who had an internal fixation in 2016/17 (*n* = 4787) instead had close-contact-casting, we estimate that CCGs could have saved approximately £778,000, £1.56million, or £2.33million respectively (assuming a saving to the NHS of £650 per patient [9]).

## Discussion

### Summary of findings

In 2016/17, nearly 20,000 people in England were admitted for management of ankle fractures at a cost of approximately £63.1million to CCGs. Incidence of ankle fractures and surgical management varied substantially by age. Despite emerging evidence that non-surgical and surgical management may achieve equivalent functional outcomes at 3-years in patients over 60 with unstable ankle fractures, the rate of surgical fixation has remained relatively stable over the decade. The health service could achieve substantial savings, at least in the short term, if a higher proportion of older patients were treated with close-contact-casting, in line with recent evidence. An increasing proportion of patients who underwent surgical fixation had intramedullary implants, although evidence that this approach is more cost-effective than extramedullary fixation or non-surgical management is insufficient.

### Strengths and limitations

We believe this is the first paper exploring ankle fracture management using routinely collected data covering the whole of England. This enabled us to analyse many patients over an extended period. Hospital reimbursement is based on the diagnosis and procedure codes recorded in HES, therefore there are incentives for these data to be comprehensively recorded. However, this is routine data so will inevitably contain data entry errors; for example, an audit between 2010 and 2013 found that 13% of primary diagnoses and 12% of primary procedures were coded incorrectly [22]. Our analysis only includes patients admitted to hospital so we cannot determine the incidence of ankle fractures managed non-surgically in the emergency department or in outpatient appointments. We also cannot distinguish between stable and unstable fractures, typically determined by analysis of radiographs or clinical assessment. We excluded non-specific lower limb fracture diagnosis codes and admissions where ankle fracture was a secondary diagnosis, so our estimates are conservative. The inclusion of the primary diagnostic code S828 would also include some more complex periarticular fractures, but the proportion of these is low and therefore not expected to affect our results [16]. Similarly, lateral malleolus avulsion fractures may be included but again numbers are expected to be small as patients wouldn’t often be admitted for such fractures (and if they were, it would primarily be for social care reasons). Although procedure codes allowed us to distinguish broad categories of management, they were not specific enough to differentiate types of intramedullary nail, such as fibular or tibio-talo-calcaneal nails, or methods of non-surgical management (e.g. cast or boot). The HES pseudonymised patient identifiers make it possible to identify readmissions, however they did not distinguish whether it was for management of the original or a new fracture or for a planned or unplanned event, therefore we inferred this based on timing.

### Comparisons to other literature

Based on analysis of UK primary care records, the annual incidence of ankle fracture is estimated to be 75 per 100,000 adults < 50 years and 104 per 100,000 50+ year olds [2]. The incidence rate in our study (44 per 100,000 adults in 2016/17) was lower, suggesting that many ankle fractures are managed in emergency and outpatient care. In Sweden (1987–2004), the incidence of hospitalisation for ankle fracture among all adults was estimated at 75 per 100,000 [23], while in Finland (1997–2006) 144 of 100,000 adults age 60+ years were hospitalised [24]. The lower rates observed in our study might be due to decreasing trends for hospitalisation in recent years, although this hypothesis is not supported by our data. Other potential explanations include differences between countries in risk factors and incidence or differences in clinician thresholds for admitting patients to hospital. Our finding that bimalleolar and trimalleolar fractures account for a substantial proportion of patients hospitalised with ankle fractures is in line with previous studies [23, 25]. The 365-day readmission rate for ankle fracture among patients with extramedullary fixations (1.0%) is lower than the previously reported all cause 30-day readmission rates (3.2%) among similar patients in the US [26].

Geographical variations in procedure rates are likely to be highest in areas of medicine where clinicians differ in their belief about the value of the procedure [27, 28]. Based on US Medicare data on patients aged 65+ years, Koval et al. [29] observed that surgical stabilisation of ankle fractures ranged from 14 to 72% across different areas of the country. We observed little geographic variation in surgical fixation rates, after adjusting for age and sex. We do not know what the ‘right’ rate of surgical fixation is, but recent evidence that close-contact-casting is more cost-effective than surgical fixation in older patients [9] indicates that reducing rates may lead to more efficient care. Results of this RCT have been reported at 3-years [8] but longer-term follow-up may be required.

### Implications for research and clinical practice

The evidence supporting surgical and non-surgical treatments of ankle fractures is relatively weak and is predominantly based on observational studies and small RCTs. The evaluation of surgical innovations provides unique challenges [30], but large well-conducted pragmatic trials are possible. In ankle fractures, the AIM trial [7–9] and recently initiated FAME trial [31], have the potential to provide more definitive answers to improve practice. Our data largely pre-date the publication of the AIM trial results; nevertheless, we did not observe any large decrease in surgical fixation rates among older patients during the trial or immediately after publication. In contrast, the DRAFFT trial demonstrating that Kirschner(K)-wires were more cost-effective than plate fixation for the management of distal radius fractures, was associated with a dramatic shift towards K-wire fixation [32]. Implementation of one surgical approach instead of another may be a simpler message to disseminate than a move from surgical to non-surgical management.

Guidelines listing close-contact-casting as a treatment option may not, in isolation, be enough to disseminate the findings of the AIM trial and reduce rates of surgical fixation in older patients. More proactive options such as CCG criteria-based access policies which define the patient subgroups where surgery will be funded, or shared decision-making tools to provide patients with a greater role in the decision making, may lead to greater changes in care delivery [33]. Our data suggest that the savings of a switch towards non-surgical care in only 25% of older patients currently treated with internal fixations would be relatively modest (~£778,000 per year), but would accumulate if sustained over time or generalised a greater proportion of patients or to other countries.

We observed small increases in the proportion of surgical fixation performed with intramedullary implants. Although intramedullary implants use smaller incisions that reduce soft tissue disruption and may allow early weight bearing, better evidence from larger trials is needed to determine their long-term cost-effectiveness [34].

## Conclusion

We found relatively stable rates of surgical fixation for ankle fractures despite emerging evidence that non-surgical and surgical management achieve equivalent long-term functional outcomes in older patients. Effective implementation of robust randomised trial findings with sufficient length of follow-up is needed to improve patient care and reduce health system costs.

## Supplementary information

**Additional file 1.** Supplementary material 1. Identifying procedures for ankle fractures.

**Additional file 2.** Supplementary material 2. Fracture management during unplanned readmissions.

## Data Availability

The data in this study were provided by patients and collected by the NHS as part of their care and support. HES data can be accessed via NHS Digital: https://digital.nhs.uk/services/data-accessrequest-service-dars.

## Abbreviations

BOAST: British Orthopaedic Association Standards for Trauma and Orthopaedics
CCG: Clinical Commissioning Group
HES: Hospital Episode Statistics
HRG: Health Resource Group
ICD10: International Classification of Diseases version 10
NHS: National Health Service
OPCS: Office of Population, Censuses and Surveys
RCT: Randomised Controlled Trial
UK: United Kingdom
