# Supplementary material for: Exploring trends in admissions and treatment for ankle fractures: a longitudinal cohort study of routinely collected hospital data in England
Source: BMC Health Serv Res. 2020 Aug 31;20:811. doi: 10.1186/s12913-020-05682-9 (PMC7457765; doi:10.1186/s12913-020-05682-9)
Supplement: Supplementary file 1 — Additional file 1. Supplementary material 1. Identifying procedures for ankle fractures. [file 12913_2020_5682_MOESM1_ESM.docx]

**Supplementary material 1**

**Identifying procedures for ankle fractures**

As people often have closed reductions and casts in outpatients, and our main focus is surgical interventions, we will include codes for both primary and secondary procedures when identifying which procedure patients had. We will use the definitions in the main paper to define whether the procedure occurred during an index visit or readmission.

**Intramedullary** fixation will be identified using the following *procedure* codes:

| W192 | Primary open reduction of fracture of long bone and fixation using rigid nail NEC |
| --- | --- |
| W193 | Primary open reduction of fracture of long bone and fixation using flexible nail |
| W194 | Primary open reduction of fracture of small bone and fixation using screw |
| W195 | Primary open reduction of fragment of bone and fixation using screw |
| W196 | Primary open reduction of fragment of bone and fixation using wire system |
| W198 | Other specified primary open reduction of fracture of bone and intramedullary fixation |
| W199 | Unspecified primary open reduction of fracture of bone and intramedullary fixation |
| W244 | Closed reduction of fracture of small bone and fixation using screw |
| W245 | Closed reduction of fragment of bone and fixation using screw |
| W231 | Secondary open reduction of fracture of bone and intramedullary fixation HFQ |

**Extramedullary** fixation will be identified using the following *procedure* codes:

| W201 | Primary open reduction of fracture of long bone and extramedullary fixation using plate NEC |
| --- | --- |
| W202 | Primary open reduction of fracture of long bone and extramedullary fixation using cerclage |
| W203 | Primary open reduction of fracture of long bone and extramedullary fixation using suture |
| W204 | Primary open reduction of fracture of long bone and complex extramedullary fixation NEC |
| W205 | Primary open reduction of fracture of ankle and extramedullary fixation NEC |
| W208 | Other specified primary open reduction of fracture of bone and extramedullary fixation |
| W209 | Unspecified primary open reduction of fracture of bone and extramedullary fixation |
| W654 | Primary open reduction of fracture dislocation of joint and internal fixation NEC |
| W232 | Secondary open reduction of fracture of bone and extramedullary fixation HFQ |
| W677 | Secondary open reduction of fracture dislocation of joint and internal fixation |

**Internal fixation (location unspecified)** will be identified using the following *procedure* codes:

| W211 | Primary reduction of intra-articular fracture of bone using arthrotomy as approach |
| --- | --- |
| W213 | Primary fixation of fragment of chondral cartilage of intra-articular fracture of bone |
| W214 | Primary intra-articular fixation of intra-articular fracture of bone NEC |
| W215 | Primary extra-articular reduction of intra-articular fracture of bone |
| W218 | Other specified primary open reduction of intra-articular fracture of bone |
| W219 | Unspecified primary open reduction of intra-articular fracture of bone |
| W242 | Closed reduction of fracture of long bone and rigid internal fixation NEC |
| W243 | Closed reduction of fracture of long bone and flexible internal fixation HFQ |
| W246 | Closed reduction of fracture of bone and fixation using nail or screw |
| W248 | Other specified closed reduction of fracture of bone and internal fixation |
| W249 | Unspecified closed reduction of fracture of bone and internal fixation |
| W281 | Application of internal fixation to bone NEC |
| W288 | Other specified other internal fixation of bone |
| W289 | Unspecified other internal fixation of bone |
| W236 | Secondary open reduction of fracture of bone and internal fixation HFQ |
| W282 | Adjustment to internal fixation of bone NEC |
| W283 | Removal of internal fixation from bone NEC |

**External fixation** will be identified using the following *procedure* codes:

| W251 | : Closed reduction of fracture of bone and fixation to skeleton HFQ |
| --- | --- |
| W258 | : Other specified closed reduction of fracture of bone and external fixation |
| W259 | : Unspecified closed reduction of fracture of bone and external fixation |
| W301 | : Application of external fixation to bone NEC |
| W308 | : Other specified other external fixation of bone |
| W309 | : Unspecified other external fixation of bone |
| W235 | : Secondary open reduction of fracture of bone and external fixation HFQ |
| W253 | : Remanipulation of fracture of bone and external fixation HFQ |
| W302 | : Adjustment to external fixation of bone NEC |
| W303 | : Removal of external fixation from bone NEC |

**‘No surgical fixation’** will be identified by the absence of **all** of the above fixations.

If more than one surgical fixation occurred within an admission, the first recorded fixation was used to categorise. As initial non-surgical stabilisation may be followed by planned or unplanned surgery, we chose to categorise patients with no surgical fixation on the index admission, but surgical fixation on readmission according to the type of surgical fixation eventually received (Figure 1).

Index admissions and any corresponding readmissions were excluded from the cohort if adjustment or removal of hardware was reported but the original fixation this related to was not. This was because in these circumstances it was unclear when the original fixation occurred (Figure 1).
