# Supplementary material for: Exploring trends in admissions and treatment for ankle fractures: a longitudinal cohort study of routinely collected hospital data in England
Source: BMC Health Serv Res. 2020 Aug 31;20:811. doi: 10.1186/s12913-020-05682-9 (PMC7457765; doi:10.1186/s12913-020-05682-9)
Supplement: Supplementary file 2 — Additional file 2. Supplementary material 2. Fracture management during unplanned readmissions. [file 12913_2020_5682_MOESM2_ESM.docx]

**Supplementary material 2**

**Fracture management during unplanned readmissions**

|  | **Initial fracture management** | | | | | | | | | | | |
| --- | --- | --- | --- | --- | --- | --- | --- | --- | --- | --- | --- | --- |
|  | **Intramedullary (n=309)** | | **Extramedullary (n=1,151)** | | **Internal unspecified (n=117)** | | **External**  **(n=179)** | | **No surgical fixation (n=1,461)** | | **Overall**  **(n=3,217)** | |
| **Management during readmission** |  |  |  |  |  |  |  |  |  |  |  |  |
| Intramedullary | 37 | 12.0% | 90 | 7.8% | 4 | 3.4% | 18 | 10.1% | 104 | 7.1% | 253 | 7.9% |
| Extramedullary | 64 | 20.7% | 257 | 22.3% | 20 | 17.1% | 50 | 27.9% | 268 | 18.3% | 659 | 20.5% |
| Internal unspecified | 15 | 4.9% | 71 | 6.2% | 7 | 6.0% | 14 | 7.8% | 53 | 3.6% | 160 | 5.0% |
| Internal adjustment | 3 | 1.0% | 11 | 1.0% | 2 | 1.7% | 0 | 0.0% | 0 | 0.0% | 16 | 0.5% |
| Internal removal | 31 | 10.0% | 137 | 11.9% | 9 | 7.7% | 5 | 2.8% | 10 | 0.7% | 192 | 6.0% |
| External | 2 | 0.6% | 12 | 1.0% | 0 | 0.0% | 14 | 7.8% | 11 | 0.8% | 39 | 1.2% |
| External adjustment | 1 | 0.3% | 2 | 0.2% | 0 | 0.0% | 7 | 3.9% | 1 | 0.1% | 11 | 0.3% |
| External removal | 6 | 1.9% | 18 | 1.6% | 3 | 2.6% | 66 | 36.9% | 2 | 0.1% | 95 | 3.0% |
| No surgical fixation | 186 | 60.2% | 651 | 56.6% | 81 | 69.2% | 77 | 43.0% | 1,063 | 72.8% | 2,058 | 64.0% |

Note. Each patient may have more than one type of fracture management during a readmission and/or more than one readmission, so column percentages may not add up to 100. Numbers and percentages stated for ‘no surgical fixation’ are those who had no fixations, adjustments or removals during any unplanned readmission.
